# A pan-cancer analysis of the oncogenic role of Golgi transport 1B in human tumors

**DOI:** 10.2478/jtim-2023-0002

**Published:** 2023-12-20

**Authors:** Bo Tian, Yanan Pang, Ye Gao, Qianqian Meng, Lei Xin, Chang Sun, Xin Tang, Yilin Wang, Zhaoshen Li, Han Lin, Luowei Wang

**Affiliations:** Department of Gastroenterology, Changhai Hospital, Naval Medical University, Shanghai 200433, China; Shanghai Institute of Pancreatic Diseases, Shanghai 200433, China; Georgetown Preparatory School, North Bethesda 20852, MD, USA

**Keywords:** pan-cancer, bioinformatics, Golgi transport 1B, prognosis, immune infiltration

## Abstract

**Background:**

Owing to the aggressiveness and treatment-refractory nature of cancer, ideal candidates for early diagnosis and treatment are needed. Golgi transport 1B (*GOLT1B*) has been associated with cellular malignant behaviors and immune responses in colorectal and lung cancer, but a systematic pan-cancer analysis on *GOLT1B* has not been conducted.

**Methods:**

The expression status and clinical association of *GOLT1B* in The Cancer Genome Atlas (TCGA) were analyzed. Genetic and methylation alterations in *GOLT1B* were explored. The relationship between *GOLT1B* and immune cell infiltration was also investigated. Genes related to *GOLT1B* expression were selected and analyzed.

**Results:**

*GOLT1B* was highly expressed in most tumors, and there was a positive correlation between *GOLT1B* expression and clinical pathological parameters. High expression levels of *GOLT1B* have been associated with poor prognosis of most cancers. Copy number amplification was the primary type of *GOLT1B* genetic alterations, which was related to the prognosis of pan-cancer cases. There were different levels of *GOLT1B* promoter methylation across cancer types. The methylation level of the probe cg07371838 and cg25816357 was closely associated with prognosis in diverse cancers. There was also a positive correlation between *GOLT1B* genetic alterations and CD4+ T lymphocytes, especially the Th2 subset, as well as between *GOLT1B* expression and the estimated infiltration value of cancer-associated fibroblasts. Serine/threonine kinase receptor-associated protein (*STRAP*), integrator complex subunit 13 (*INTS13*), and ethanolamine kinase 1 (*ETNK1*) were the most relevant genes for *GOLT1B* expression, and their interactions with *GOLT1B* were involved in regulating the transforming growth factor (TGF)-β receptor signaling pathway and epithelial-mesenchymal transition (EMT).

**Conclusions:**

This pan-cancer analysis provided a comprehensive understanding of the oncogenic role of *GOLT1B*, highlighting a potential mechanism whereby *GOLT1B* influences the tumor microenvironment, as well as cancer immunotherapy.

## Introduction

Globally, cancer is one of the primary causes of morbidity and mortality. Oncogenesis is a multistep and multilayered process, in which initiation and development are concomitant with aberrant biological activities of multiple proteins and lipids. Changeable structure and regulatory diversity of these functional molecules pose great challenges when exploring valuable prognostic biomarkers and effective therapeutic targets.^[1,2]^ As a central hub in trafficking, sorting, and modifying proteins and lipids, the Golgi apparatus (GA) plays a major role in maintaining cellular homeostasis while regulating cell differentiation and development.^[3]^ This organelle is characterized by a dynamic stacked ribbon-like structure and a complex multi-compartment system. Based on the sophisticated constitution, the fine-tuned regulatory mechanism of GA ensures efficacy and accuracy in its normal biological functions, such as post-translational protein modification, vesicular transportation, and cisternal maturation.^[4,5]^ Structural changes and functional disorganization of GA contribute to a number of pathophysiological changes, including oncogenesis.^[6]^ These disorders include Golgi fragmentation, aberrant Golgi glycosylation, and membrane trafficking perturbations resulting in disrupted Golgi pH homeostasis, which are frequently related to mutations in Golgi resident proteins.^[7,8]^

Membrane trafficking defects resulting from anomalous changes in resident proteins are closely associated with cell signaling communication, dissociation and invasion, immune regulation, and metastasis in cancer.^[9]^ Golgi transport 1B (*GOLT1B*) is a member of the GOT1 family, and abnormal expressions of proteins in this family are associated with GA membrane trafficking disorders and cellular malignant behaviors.^[10]^ The GOT1 family consists of two primary members, *GOLT1A* and *GOLT1B*. High expression of *GOLT1A* has been correlated with poor prognosis and resistance to endocrine therapy in breast cancer.^[11]^ The *GOLT1A-KISS1* fusion transcript was shown to be a biomarker of adenoid cystic carcinoma metastasis.^[12]^ *GOLT1B* is located primarily in early Golgi cisternae, the absence of which induces a substantial reduction in the endoplasmic reticulum (ER)-Golgi transport efficiency.^[13,14]^ The amplification of *GOLT1B* is correlated with poor prognosis in lung cancer.^[15]^ *GOLT1B* contributes to epithelial-mesenchymal transition (EMT) in colorectal cancer (CRC) by activating WNT signaling, influencing the secretion of interferon (IFN)-γ, and apoptosis of tumor-infiltrating T lymphocytes.^[16, 17]^ Nevertheless, previous studies have only assessed *GOLT1B* in selected types of cancer, and the potential mechanism by which *GOLT1B* promotes tumorigenesis in divergent tumor types remains unclear.

Pan-cancer analyses enable the investigation of the differential expression of target genes and the corresponding abnormal regulatory mechanisms essential for tumorigenesis and progression across multiple layers of alterations.^[18]^ To our knowledge, this is the first pan-cancer study regarding the oncogenic role of *GOLT1B* by comprehensively assessing gene expression, clinical association, survival status, DNA methylation, genetic alterations, immune infiltration, and relevant molecular mechanisms.

## Methods

### Oncomine

The mRNA levels of *GOLT1B* in diverse cancer types were analyzed in Oncomine, a publicly available online database providing genome-wide expression analysis with cancer microarray information.^[19]^ In the present study, the statistical significance thresholds were set to *P* < 0.01, fold change of 1.5, and gene rank in the top 10%. The difference in the expression of *GOLT1B* between cancer and normal tissues was analyzed using Student’s t-test.

### Tumor immune estimation resource, version 2

Tumor immune estimation resource, version 2 (TIMER2) is a web tool for the systematic evaluation of gene expression differences, the correlation with immune cell infiltration, as well as relevant clinical impacts.^[20]^ We input *GOLT1B* in the “Gene_DE” module of TIMER2 and further observed the expression difference of *GOLT1B* between tumor and adjacent normal tissues for the specific tumor subtypes of the TCGA project. Additionally, we used the “immune association” module of TIMER2 to explore the mutation frequency and somatic copy number alteration (sCNA) status of *GOLT1B* across multiple cancers by inputting *GOLT1B* in the “Mutation” and “sCNA” boxes. We applied the “Immune-Gene” module of the TIMER2 web server to explore the association between *GOLT1B* expression and immune infiltration across all TCGA tumors.

### Gene Expression Profiling Interactive Analysis 2

The Gene Expression Profiling Interactive Analysis 2 (GEPIA2) database is used for cancer and normal gene expression and interactive analysis based on genotype-tissue expression (GTEx) and TCGA data.^[21]^ We used the “expression analysis box plots” module of the GEPIA2 to obtain box plots of the expression difference between these tumor tissues and the corresponding normal tissues of the GTEx database, under the settings of *P*-value cutoff = 0.01, log^2^fold change (FC) cutoff = 1, and “Match TCGA normal and GTEx data.” We obtained violin plots of the *GOLT1B* expression in different pathological stages of all TCGA tumors *via* the “pathological stage plot” module of GEPIA2. Moreover, the “survival map” module of GEPIA2 was applied to obtain the OS and DFS significance map data of *GOLT1B*. The log-rank test was used and the survival plots were also obtained through the “survival analysis” module of GEPIA2. Furthermore, we applied the “correlation analysis” module of GEPIA2 to perform a pairwise gene Pearson correlation analysis of *GOLT1B* and selected genes.

### The University of ALabama at Birmingham CANcer data analysis Portal (UALCAN)

The UALCAN database is an online resource for gene analysis based on OMICS data (TCGA, MET500, and Clinical proteomic tumor analysis consortium [CPTAC]).^[22]^ In our study, UALCAN was used to investigate the associations between the protein expression of *GOLT1B* and cancer-related clinical factors. We used the CPTAC data set in the UALCAN portal to explore the protein expression level of *GOLT1B* between primary tumor and normal tissues by entering “*GOLT1B*.” The promoter methylation level of *GOLT1B* was also analyzed by applying the “expression analysis box plots” module. Statistical differences were assessed by using Student’s t-test, and the statistical significance threshold was set at *P* < 0.05.

### cBioPortal

cBioPortal is an online resource with visual and multidimensional cancer genomic data.^[23,24]^ We chose the “TCGA Pan-Cancer Atlas Studies” in the “Quick select” section and entered “*GOLT1B*” for queries of the genetic alteration characteristics of *GOLT1B*. The “plots” module was used to analyze the correlation of *GOLT1B* mRNA and *GOLT1B* Log2 copy-number values. The results of the alteration frequency, mutation type, and sCNA across all TCGA tumors were explored in the “cancer types summary” module. Additionally, we used the “comparison/survival” module to obtain the data on the overall, disease-free, progression-free, and disease-free survival differences for the TCGA cancer cases with or without *GOLT1B* genetic alteration. Kaplan-Meier plots with log-rank *P*-value were obtained as well. The cellular pathways of *GOLT1B* genetic alteration were explored in the “pathways” module.

### MethSurv

MethSurv, a web tool for assessing DNA methylation, expression, and clinical data visualization.^[25]^ This tool was used to perform the survival analysis by establishing Cox proportional hazards models for CpGs located in or around the proximity of the targeted genes across different types of cancer. We entered *GOLT1B* in the searching box in “all cancers” module to perform survival analysis for a CpG located in or around the proximity of *GOLT1B*. In order to evaluate the differences in survival analysis, the methylation levels of patients were divided into higher (the methylation β value higher than the cut-off point) and lower groups (the methylation β value lower than the cut-off point). *P* < 0.05 was considered statistically significant.

### String

STRING is a protein interaction website aiming to establish a global network of unique computational predictions.^[26]^ We searched the STRING website by using the query of a single protein name (“*GOLT1B*”) and organism (“Homo sapiens”). Subsequently, we set the following main parameters: minimum required interaction score (“low confidence [0.150]”), meaning of network edges (“evidence”), max number of interactors to show (“no more than 50 interactors” in 1st shell), and active interaction sources (“experiments”). Finally, the available experimentally determined *GOLT1B*-binding proteins were obtained, and protein–protein interaction network was performed.

### GeneMANIA

GeneMANIA contains information on genes and prioritizes genes for functional assays with a highly accurate prediction algorithm.^[27]^ We input *GOLT1B*-related genes in the searching box of GeneMANIA and obtained the protein–protein interaction network map and relevant functional assays in the “functions data” module.

### Kyoto encyclopedia of genes and genomes/gene ontology analysis

The “similar gene detection” module of GEPIA2 was used to obtain the top 100 *GOLT1B*-correlated genes. We combined the two sets of genes (GEPIA2 and STRING) to perform Kyoto encyclopedia of genes and genomes (KEGG) pathway analysis and gene ontology (GO) enrichment analysis. The KEGG pathway and GO analyses were performed to determine the pan-cancer biological and molecular functions of *GOLT1B* by virtue of the R packages of ClusterProfiler and org.Hs.eg.db. The R package of ggplot2 was used to visualize the enriched pathways.

## Results

### The pan-cancer expression profiles of GOLT1B

**Figure 1:**
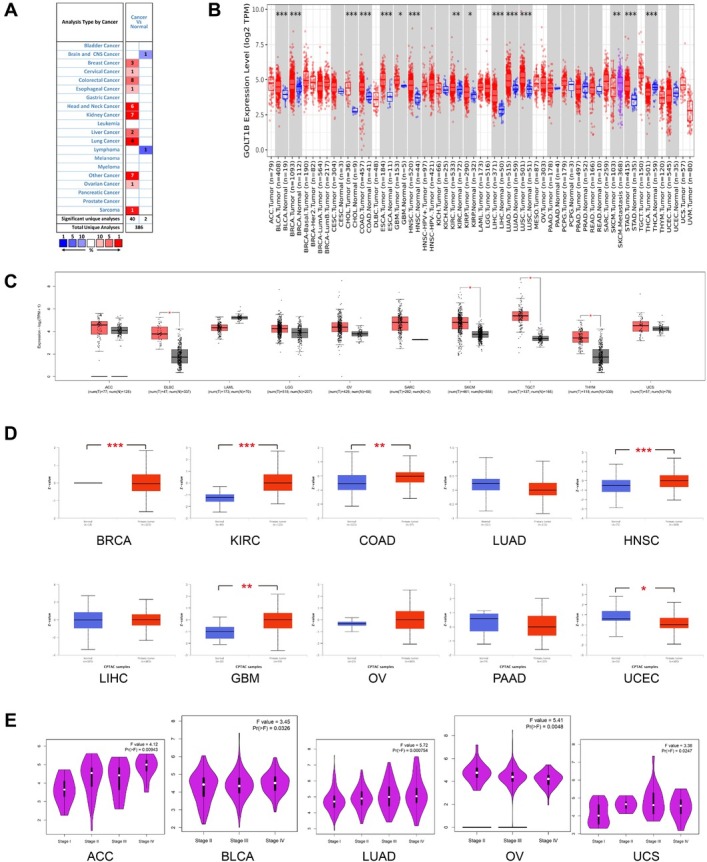
The expression of GOLT1B in multiple cancers. (A) Increased (red) or decreased (blue) expression of GOLT1B in different cancer tissues, compared with normal tissues in Oncomine. The number in each grid represents the amount of datasets. (B) Expression levels of GOLT1B in different cancer types from TCGA data in TIMER2. (C) Expression difference between the tumor tissues and normal tissues of the GTEx (tumors without normal tissues in TCGA). (D) The expression level of GOLT1B total protein in cancers of CPTAC data set. *P < 0.05,**P < 0.01. (E) Based on the TCGA data, the expression levels of the GOLT1B gene were analyzed by the main pathological stages (stages I, II, III, and IV) of ACC, BLCA, LUAD, OV, and UCS. Log_2_(TPM +1) was applied for the log scale. ACC: adrenocortical carcinoma; BLCA: bladder urothelial carcinoma; BRCA: breast invasive carcinoma, CESC: cervical squamous cell carcinoma and endocervical adenocarcinoma; CHOL: cholangiocarcinoma; COAD: colon adenocarcinoma, DLBC: lymphoid neoplasm diffuse large B-cell lymphoma; ESCA: esophageal carcinoma; GBM: glioblastoma multiforme; HNSC: head and neck squamous cell carcinoma; KICH: kidney chromophobe; KIRC: kidney renal clear cell carcinoma; KIRP: kidney renal papillary cell carcinoma; LAML: acute myeloid leukemia; LGG: brain lower grade glioma; LIHC: liver hepatocellular carcinoma; LUAD: lung adenocarcinoma; LUSC: lung squamous cell carcinoma; MESO: mesothelioma; OV: ovarian serous cystadenocarcinoma; PAAD: pancreatic adenocarcinoma; PCPG: pheochromocytoma and paraganglioma; PRAD: prostate adenocarcinoma; READ: rectum adenocarcinoma; SARC: sarcoma; SKCM: skin cutaneous melanoma; STAD: stomach adenocarcinoma; TGCT: testicular germ cell tumors; THCA: thyroid carcinoma; THYM: thymoma; UCEC: uterine corpus endometrial carcinoma; UCS: uterine carcinosarcoma; UVM: uveal melanoma.

*GOLT1B* mRNA expression levels in different types of cancer were analyzed in Oncomine (Figure 1A). The pooled analysis confirmed that, compared with normal tissues, the transcriptional levels of *GOLT1B* were significantly elevated in tumor tissues. TIMER2 results also demonstrated that the expression levels of *GOLT1B* were upregulated in the tumor tissues of 15 cancer types, including bladder urothelial carcinoma (BLCA), cholangiocarcinoma (CHOL), breast invasive carcinoma (BRCA), colon adenocarcinoma (COAD), esophageal carcinoma (ESCA), head and neck squamous cell carcinoma (HNSC), glioblastoma multiforme (GBM), kidney renal clear cell carcinoma (KIRC), kidney renal papillary cell carcinoma (KIRP), liver hepatocellular carcinoma (LIHC), lung squamous cell carcinoma (LUSC), lung adenocarcinoma (LUAD), prostate adenocarcinoma (PRAD), stomach adenocarcinoma (STAD), and thyroid carcinoma (THCA) (Figure 1B). Using normal tissues as the controls, the expression of *GOLT1B* was found to be upregulated in the GTEx data set for diffuse large B-cell lymphoma (DLBC), skin cutaneous melanoma (SKCM), testicular germ cell tumors (TGCT), and thymoma (THYM) tissues (Figure 1C). However, there was no significant difference in *GOLT1B* expression between tumor and normal tissues for adrenocortical carcinoma (ACC), acute myeloid leukemia (LAML), brain lower grade glioma (LGG), ovarian serous cystadenocarcinoma (OV), sarcoma (SARC), or uterine carcinosarcoma (UCS).

Furthermore, the protein expression profiles of *GOLT1B* in various tumor and normal tissues were assessed using the CPTAC database. CPTAC results showed that the protein expression of *GOLT1B* was higher in KIRC, COAD, HNSC, and GBM primary tissues, and lower in BRCA and UCEC primary tissues compared to the corresponding normal tissues (Figure 1D). The correlation between *GOLT1B* mRNA expression and the pathological stage of cancers including ACC, BLCA, LUAD, OV, and UCS was obtained (Figure 1E, *P* < 0.05). The box plots showed that higher levels of *GOLT1B* expression were associated with higher tumor stages in ACC, BLCA, LUAD, and UCS. The opposite expression trend was observed in OV.

### Correlation of GOLT1B expression with prognosis

**Figure 2:**
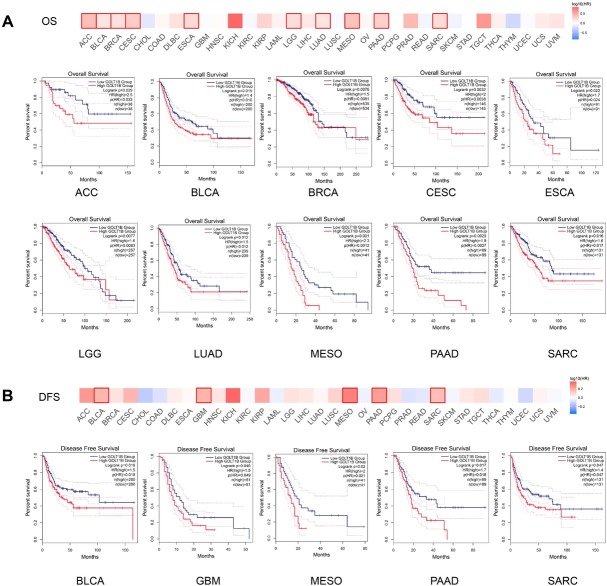
Prognostic significance of GOLT1B expression in many cancers. Comparison of OS (A) and DFS (B) between high and low expression of GOLT1B in different cancer types in GEPIA2. The survival map and Kaplan-Meier curves with positive results are given. OS: overall survival; DFS: disease-free survival; ACC: adrenocortical carcinoma; BLCA: bladder urothelial carcinoma; BRCA: breast invasive carcinoma, CESC: cervical squamous cell carcinoma and endocervical adenocarcinoma; CHOL: cholangiocarcinoma; COAD: colon adenocarcinoma, DLBC: lymphoid neoplasm diffuse large B-cell lymphoma; ESCA: esophageal carcinoma; GBM: glioblastoma multiforme; HNSC: head and neck squamous cell carcinoma; KICH: kidney chromophobe; KIRC: kidney renal clear cell carcinoma; KIRP: kidney renal papillary cell carcinoma; LAML: acute myeloid leukemia; LGG: brain lower grade glioma; LIHC: liver hepatocellular carcinoma; LUAD: lung adenocarcinoma; LUSC: lung squamous cell carcinoma; MESO: mesothelioma; OV: ovarian serous cystadenocarcinoma; PAAD: pancreatic adenocarcinoma; PCPG: pheochromocytoma and paraganglioma; PRAD: prostate adenocarcinoma; READ: rectum adenocarcinoma; SARC: sarcoma; SKCM: skin cutaneous melanoma; STAD: stomach adenocarcinoma; TGCT: testicular germ cell tumors; THCA: thyroid carcinoma; THYM: thymoma; UCEC: uterine corpus endometrial carcinoma; UCS: uterine carcinosarcoma; UVM: uveal melanoma.

The correlation between *GOLT1B* expression levels and prognosis in different cancers was analyzed by using GEPIA2. The cutoff-high (50%) and cutoff-low (50%) values were used as the expression thresholds for splitting the high-expression and low-expression cohorts. As shown in Figure 2A, high *GOLT1B* expression was associated with poor overall survival (OS) for ACC, BLCA, BRCA, CESC, ESCA, LGG, LUAD, MESO, PAAD, and SARC within the TCGA data set. Additionally, there were significant correlations between high *GOLT1B* expression and poor disease-free survival (DFS) for TCGA cases of BLCA, GBM, MESO, PAAD, and SARC (Figure 2B).

### Genetic alteration analysis of GOLT1B

**Figure 3:**
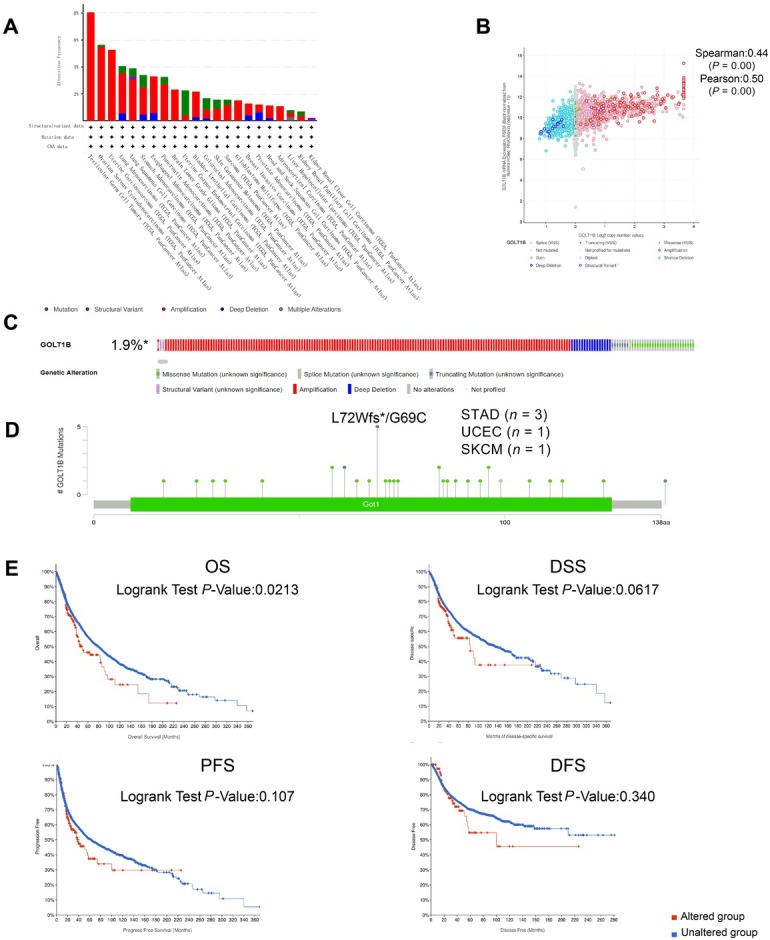
Genetic alteration analysis of GOLT1B in cBioportal. (A) Alteration frequency of GOLT1B in different cancers. (B) GOLT1B Log2 copy number with mRNA expression. (C–D) Types, sites, and case number of GOLT1B and genetic alteration of GOLT1B. (E) Association between genetic alteration of GOLT1B and the clinical survival prognosis of pan-cancer. OS: overall survival; DSS: disease-specific survival; PFS: progression-free survival; DFS: disease-free survival; STAD: stomach adenocarcinoma; UCEC: uterine corpus endometrial carcinoma; SKCM: skin cutaneous melanoma.

The genetic alteration status of *GOLT1B* in different tumor samples from TCGA cohorts was investigated by applying cBioPortal. As shown in Figure 3A, the highest alteration frequency of *GOLT1B* (> 8%) was observed in patients with non-seminomatous germ cell tumors, with amplification as a unique alteration type. Copy number amplification was the primary type of *GOLT1B* genetic alterations, with an alteration frequency of approximately 1.9% in pan-cancer types (Figure 3C). There was a positive correlation between *GOLT1B* Log2 copy number and *GOLT1B* mRNA expression (Figure 3B). The types, sites, and case numbers of *GOLT1B* genetic alterations are presented in Figure 3C. Missense mutation of *GOLT1B* is the main type of genetic mutations. The L72Wfs*6/G69C frameshift deletion alteration was detected in three cases of STAD, one case of UCEC, and one case of SCKM (Figure 3D), which could induce a frameshift mutation in the *GOLT1B* gene.

We further explored the association between genetic alterations in *GOLT1B* and the clinical survival prognosis of pan-cancer cases. Cases with *GOLT1B* genetic alterations showed significantly poorer OS (*P* = 0.0213) but not disease-specific survival (DSS) (*P* = 0.0617), DFS (*P* = 0.107), or progression-free survival (PFS) (*P* = 0.340), compared with cases without *GOLT1B* alterations (Figure 3E).

### Methylation alteration analysis of GOLT1B

**Figure 4:**
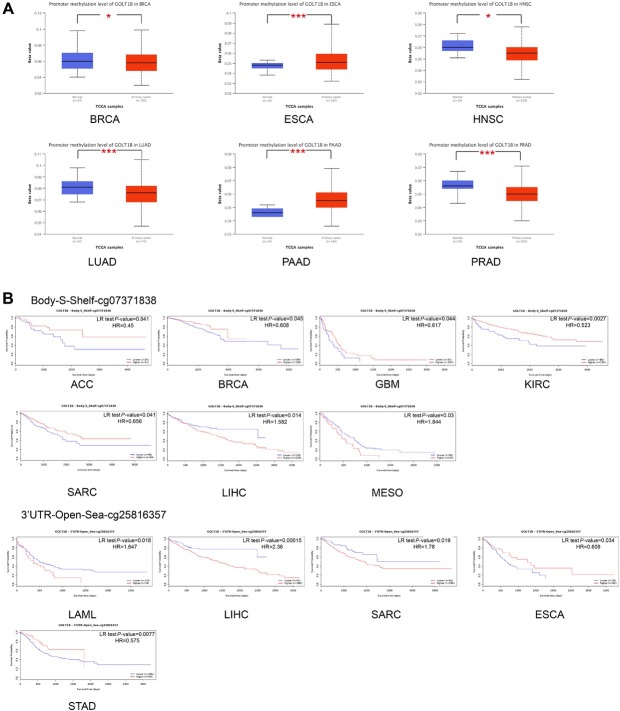
Methylation alteration analysis of GOLT1B. (A) Promoter methylation level of GOLT1B in multiple tumors *via* the UALCAN. (B) Analysis of the relationships among GOLT1B methylation levels at specific probes (cg07371838 and cg25816357) and survival time in MethSurv. ACC: adrenocortical carcinoma; BRCA: breast invasive carcinoma, ESCA: esophageal carcinoma; GBM: glioblastoma multiforme; HNSC: head and neck squamous cell carcinoma; KIRC: kidney renal clear cell carcinoma; LAML: acute myeloid leukemia; LIHC: liver hepatocellular carcinoma; LUAD: lung adenocarcinoma; MESO: mesothelioma; PAAD: pancreatic adenocarcinoma; PRAD: prostate adenocarcinoma; SARC: sarcoma; STAD: stomach adenocarcinoma. The meaning of * is " *P < 0.05, **P < 0.01.

We used the methylation module of UALCAN to investigate the potential association between *GOLT1B* DNA methylation and the pathogenesis of different tumors in the TCGA dataset. The level of promoter methylation was significantly lower in the primary tissues of BRCA, HNSC, LUAD, and PRAD but was higher in the primary tissues of ESCA and PAAD, compared to those in the corresponding normal tissues (Figure 4A). The potential association between *GOLT1B* DNA methylation sites and the prognosis of multiple cancers was investigated by using the MethSurv approach. The correlation between the methylation level of probe cg07371838 and cg25816357 of *GOLT1B* and the prognosis of multiple cancer cases was shown in Figure 4B. The Kaplan-Meier plots in the MethSurv database showed that as for cg07371838, higher methylation level in ACC, BRCA, GBM, KIRC, and SARC cases and lower methylation level in LIHC and MESO cases were associated with poorer prognosis. As for cg25816357, the higher methylation level in ESCA and STAD cases and lower methylation level in LAML, LIHC, and SARC cases were linked with poorer prognosis.

### Association between immune infiltration levels and GOLT1B genetic alterations

**Figure 5:**
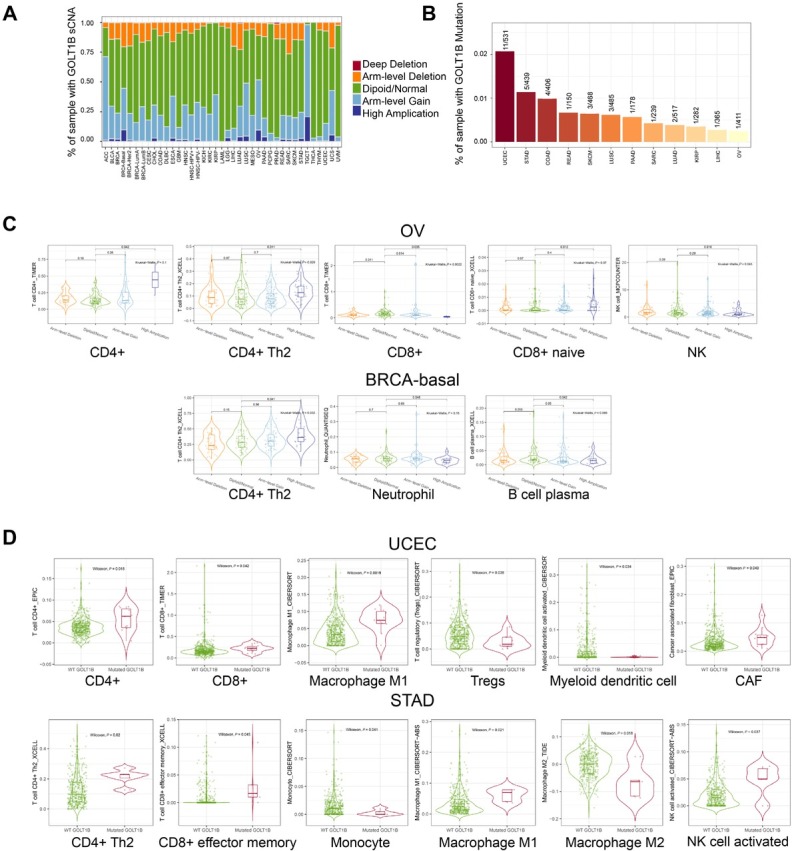
Association between immune infiltration level and GOLT1B genetic alterations. (A) Somatic copy number alteration (sCNA) states of GOLT1B for all TCGA cancer types. (B) Mutation frequency of GOLT1B for each TCGA cancer type. (C) Differential immune infiltration among different sCNA status of GOLT1B in OV and BRCA-basal. (D) Differential immune infiltration in the mutated vs wild-type tumors of GOLT1B in UCEC and STAD. ACC: adrenocortical carcinoma; BLCA: bladder urothelial carcinoma; BRCA: breast invasive carcinoma, CESC: cervical squamous cell carcinoma and endocervical adenocarcinoma; CHOL: cholangiocarcinoma; COAD: colon adenocarcinoma, DLBC: lymphoid neoplasm diffuse large B-cell lymphoma; ESCA: esophageal carcinoma; GBM: glioblastoma multiforme; HNSC: head and neck squamous cell carcinoma; KICH: kidney chromophobe; KIRC: kidney renal clear cell carcinoma; KIRP: kidney renal papillary cell carcinoma; LAML: acute myeloid leukemia; LGG: brain lower grade glioma; LIHC: liver hepatocellular carcinoma; LUAD: lung adenocarcinoma; LUSC: lung squamous cell carcinoma; MESO: mesothelioma; OV: ovarian serous cystadenocarcinoma; PAAD: pancreatic adenocarcinoma; PCPG: pheochromocytoma and paraganglioma; PRAD: prostate adenocarcinoma; READ: rectum adenocarcinoma; SARC: sarcoma; SKCM: skin cutaneous melanoma; STAD: stomach adenocarcinoma; TGCT: testicular germ cell tumors; THCA: thyroid carcinoma; THYM: thymoma; UCEC: uterine corpus endometrial carcinoma; UCS: uterine carcinosarcoma; UVM: uveal melanoma.

The potential relationship between the different levels of immune infiltration and *GOLT1B* genetic alterations in diverse cancer types in TCGA was investigated using the TIMER2. The sCNA state and mutation frequency of *GOLT1B* for each cancer type was shown in Figure 5A and 5B. The correlation between the infiltration level of different immune cells and *GOLT1B* genetic mutations was also assessed. As shown in Figure 5C, there were significant correlations between the copy number of sCNA and different immune infiltration levels in OV and BRCA, which possessed appropriate alternation samples of diverse CNA states for comparison. We also explored the difference in immune cell infiltration between the mutated and wild-type tumors of *GOLT1B* in UCEC and STAD (Figure 5D). A positive association between *GOLT1B* genetic alterations and CD4+ T lymphocytes, especially the Th2 subset, was observed (Figure 5C and 5D).

### Association between immune infiltration levels and GOLT1B expression

**Figure 6:**
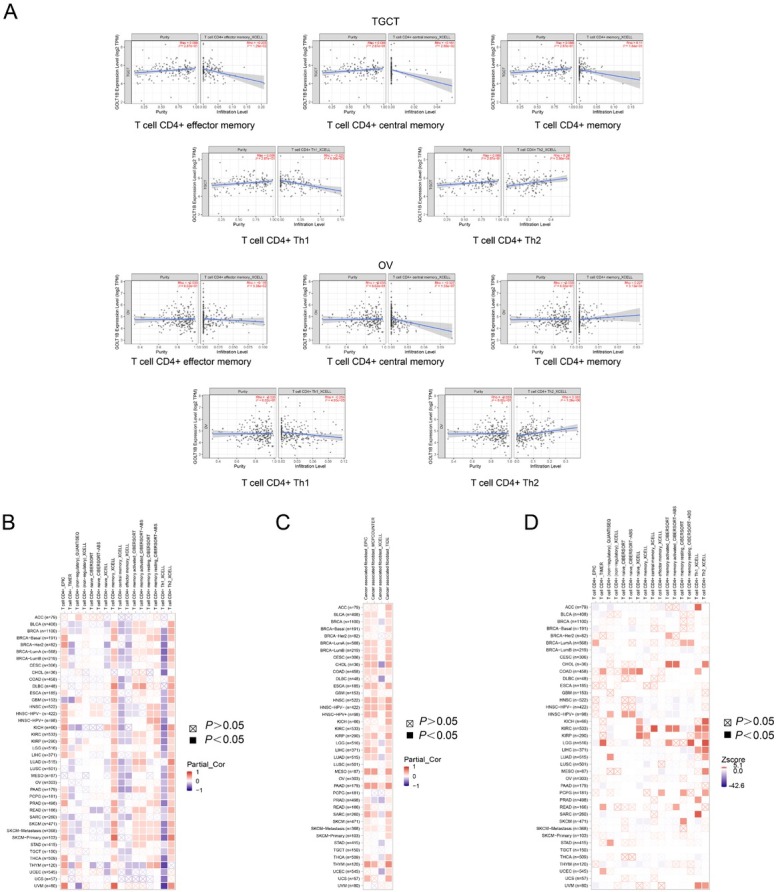
Association between immune infiltration level and GOLT1B expression. (A) The potential correlation between the expression level of the GOLT1B gene and the infiltration level of T cell CD4+ diverse subsets in TGCT and OV. (B) Heatmap of correlation: GOLT1B expression and infiltration value of T cell CD4+cell. (C) Heatmap of correlation: GOLT1B expression and infiltration value of cancer-associated fibroblasts for the TCGA tumors. (D) Heatmap of prognosis: GOLT1B expression and the infiltration level of T cell CD4+ for the TCGA tumors. ACC: adrenocortical carcinoma; BLCA: bladder urothelial carcinoma; BRCA: breast invasive carcinoma, CESC: cervical squamous cell carcinoma and endocervical adenocarcinoma; CHOL: cholangiocarcinoma; COAD: colon adenocarcinoma, DLBC: lymphoid neoplasm diffuse large B-cell lymphoma; ESCA: esophageal carcinoma; GBM: glioblastoma multiforme; HNSC: head and neck squamous cell carcinoma; KICH: kidney chromophobe; KIRC: kidney renal clear cell carcinoma; KIRP: kidney renal papillary cell carcinoma; LAML: acute myeloid leukemia; LGG: brain lower grade glioma; LIHC: liver hepatocellular carcinoma; LUAD: lung adenocarcinoma; LUSC: lung squamous cell carcinoma; MESO: mesothelioma; OV: ovarian serous cystadenocarcinoma; PAAD: pancreatic adenocarcinoma; PCPG: pheochromocytoma and paraganglioma; PRAD: prostate adenocarcinoma; READ: rectum adenocarcinoma; SARC: sarcoma; SKCM: skin cutaneous melanoma; STAD: stomach adenocarcinoma; TGCT: testicular germ cell tumors; THCA: thyroid carcinoma; THYM: thymoma; UCEC: uterine corpus endometrial carcinoma; UCS: uterine carcinosarcoma; UVM: uveal melanoma.

The associations between the immune infiltration level of CD4+ cells and cancer-associated fibroblasts with *GOLT1B* expression were explored using TIMER2. There was a significant correlation between the CD4+ subsets of T cells and *GOLT1B* expression in TGCT and OV, two cancer types with the highest alteration frequency of copy number of sCNA (Figure 6A). Meanwhile, data from xCell revealed that the level of CD4+ memory T cell subset infiltration was positively associated with *GOLT1B* expression in pan-cancer analyses, while the level of central and effector memory subset infiltration was negatively associated with *GOLT1B* expression (Figure 6B). Results of the CIBERSORT and CIBERSORT-ABS algorithms showed that there were positive correlations between activated CD4+ memory T cells, resting subsets, and *GOLT1B* expression in most cancer types such as BRCA and SKCM (Figure 6B). In addition, the heatmap of all algorithms showed a positive correlation between *GOLT1B* expression and the estimated infiltration value of cancer-associated fibroblasts for most TCGA tumors, including COAD, HNSC, HNSC-HPV, and PAAD (Figure 6C). Moreover, we investigated the association between the infiltration level of different CD4+ T cell subsets and clinical prognosis across different cancer types. As shown in Figure 6D, a high infiltration level of the Th2 subset was correlated with poor prognosis of tumors with the largest number.

### Interactions and relevant pathways of GOLT1B-related genes

**Figure 7:**
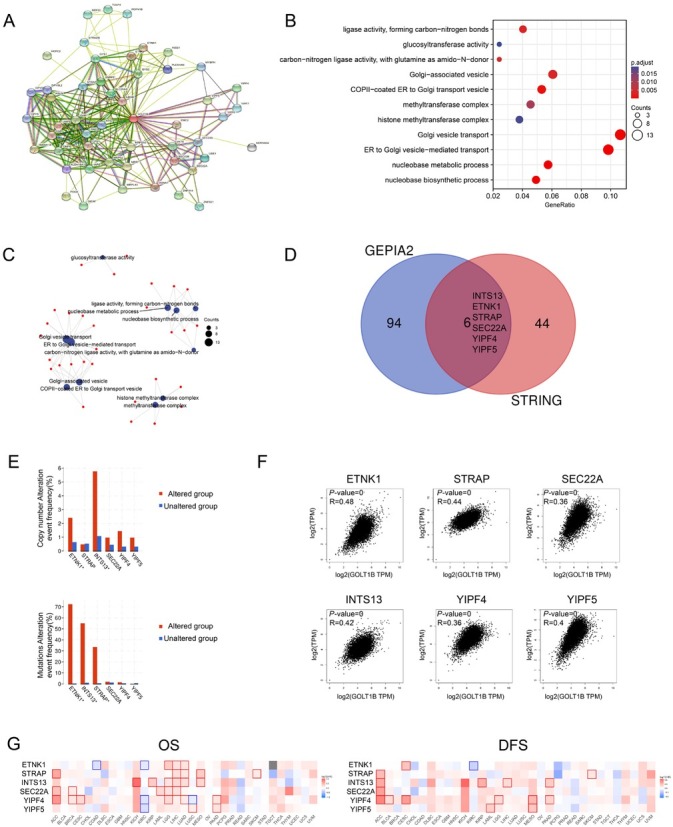
Interactions and relevant pathways of GOLT1B-related genes. (A) Network of genes for targeting genes from STRING. (B) Bubble chart of KEGG pathway analysis for targeting genes from STRING and GEPIA2. (C) The cnetplot for the molecular function data in GO analysis. (D) Intersection genes of 50 GOLT1B-binding and interacted genes and top 100 GOLT1B-correlated genes from GEPIA2. (E) Genetic alteration frequency of six relevant genes in GOLT1B mutation alteration tumor groups of TCGA tumor. (F) Analysis of the expression correlation between GOLT1B and intersection genes in TCGA cancers of GEPIA2. (G) Survival maps of comparing high and low expression of relevant genes among different cancer types in GEPIA2. OS: overall survival; DFS: disease-free survival; ACC: adrenocortical carcinoma; BLCA: bladder urothelial carcinoma; BRCA: breast invasive carcinoma, CESC: cervical squamous cell carcinoma and endocervical adenocarcinoma; CHOL: cholangiocarcinoma; COAD: colon adenocarcinoma, DLBC: lymphoid neoplasm diffuse large B-cell lymphoma; ESCA: esophageal carcinoma; GBM: glioblastoma multiforme; HNSC: head and neck squamous cell carcinoma; KICH: kidney chromophobe; KIRC: kidney renal clear cell carcinoma; KIRP: kidney renal papillary cell carcinoma; LAML: acute myeloid leukemia; LGG: brain lower grade glioma; LIHC: liver hepatocellular carcinoma; LUAD: lung adenocarcinoma; LUSC: lung squamous cell carcinoma; MESO: mesothelioma; OV: ovarian serous cystadenocarcinoma; PAAD: pancreatic adenocarcinoma; PCPG: pheochromocytoma and paraganglioma; PRAD: prostate adenocarcinoma; READ: rectum adenocarcinoma; SARC: sarcoma; SKCM: skin cutaneous melanoma; STAD: stomach adenocarcinoma; TGCT: testicular germ cell tumors; THCA: thyroid carcinoma; THYM: thymoma; UCEC: uterine corpus endometrial carcinoma; UCS: uterine carcinosarcoma; UVM: uveal melanoma; KEGG: Kyoto encyclopedia of genes and genomes; GOLT1B: golgi transport 1B.

To further investigate the molecular mechanism of *GOLT1B* in tumorigenesis, we screened *GOLT1B*-binding proteins and genes related to *GOLT1B* expression using pathway enrichment analyses. A total of 50 *GOLT1B*-binding proteins supported by experimental evidence were obtained using the STRING tool. The interaction network of these proteins is shown in Figure 7A. After combining all tumor expression data from TCGA using the GEPIA2 tool, the top 100 genes associated with *GOLT1B* expression were selected. GO and KEGG pathway analyses of the above targeting genes from STRING and GEPIA2, Golgi vesicle transport, and ER to Golgi vesicle-mediated transport were implicated in the effect of *GOLT1B* on cancer pathogenesis (Figure 7B and 7C). Venn analysis of the above two groups of target genes identified six intersection genes including integrator complex subunit 13 (*INTS13*), ethanolamine kinase 1 (*ETNK1*), serine/threonine kinase receptor-associated protein (*STRAP*), *SEC22A*, *YIPF4*, and *YIPF5* (Figure 7D), all of which were positively correlated with *GOLT1B* expression (Figure 7F). The corresponding heatmap also showed positive correlations between *GOLT1B* and the above six genes in most cancer types (Supplementary Figure 1A). Furthermore, the primary oncogenic roles of the six intersection genes in most TCGA cancer types were observed in the OS and DFS prognosis maps (Figure 7G).

### Association of intersection genes with prognosis and GOLT1B genetic alterations

To determine the underlying molecular mechanism of the interaction, we investigated whether the alteration frequencies of intersection genes were associated with *GOLT1B*. Both *ETNK1* and INST13 showed statistically significant differences in *GOLT1B* sCNA and mutation groups, whereas *STRAP* was only identified in the *GOLT1B* sCNA group (Figure 7E). The top 20 genes with the highest alteration frequencies in *GOLT1B* sCNA and mutation groups are shown in Supplementary Figure 1B and 1C. Especially, *TP53* had the highest alteration frequency in the *GOLT1B* altered group. The genetic alteration types of *STRAP*, *INTS13*, and *ETNK1* were further explored. High amplification was identified as the primary sCNA of each gene, and missense mutations as the primary mutation type for *INTS13* and *ETNK1* (Supplementary Figure 1D). Additionally, the associations between genetic alterations of the three genes and prognosis were analyzed (Supplementary Figure 1E). Compared with cases without *INTS13* genetic alteration, cases with *INTS13* genetic alteration were associated with poor prognosis in DSS and PFS, but not in OS or DFS.

### Interaction network of relevant genes and cellular pathways of GOLT1B genetic alteration

**Figure 8:**
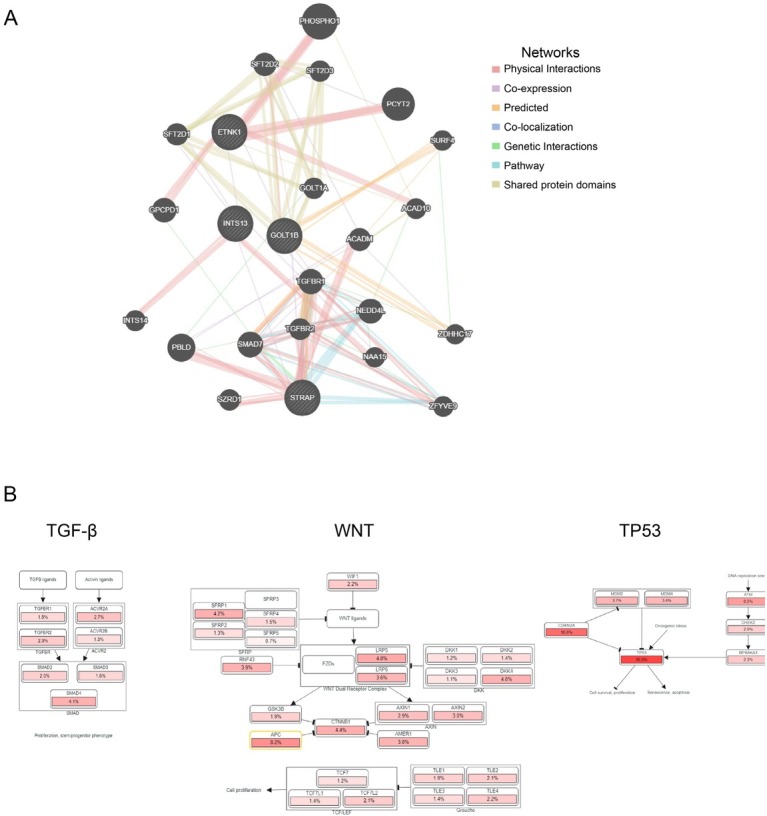
Interaction network of relevant genes and cellular pathways of GOLT1B genetic alteration. (A) Protein–protein interaction network of relevant genes from GeneMANIA. (B) Cellular pathways of GOLT1B genetic alteration. TGF: transforming growth factor; GOLT1B: golgi transport 1B.

To analyze the interaction patterns of *GOLT1B*, *STRAP*, *INTS13*, and *ETNK1* as well as the functions of associated molecules, a PPI network was constructed using GeneMANIA (Figure 8A). Functional assays showed that the corresponding molecules were primarily related to the regulation of the transforming growth factor (TGF)-β receptor signaling pathway and EMT according to false discovery rate (FDR) (Supplementary Table 1). With the highest relevant ranks in cBioportal, WNT, *TP53*, and TGF-β pathways and their primary genetic alterations were shown in Figure 8B.

## Discussion

Owing to the aggressiveness, late diagnosis, and treatment-refractory nature of cancer, ideal candidates for biomarkerrelated early diagnosis and treatment are still being explored. The multiple functions of GA and its centrality in the intersection between exocytic and endocytic membrane trafficking routes provide potential targets for cancer diagnosis and therapy.^[7]^ Diverse GA-related molecules participate in tumor development and progression *via* multilayered mechanisms and pathways.^[28,29]^ The present study is the first to comprehensively examine the expression of *GOLT1B* in a pan-cancer data set. We found that the mRNA expression of *GOLT1B* was upregulated in most cancer tissues and was associated with a poor prognosis of multiple cancers. Moreover, *GOLT1B* was positively correlated with the Th2 subset of CD4+ cells and cancer-associated fibroblasts, playing a specific role in immune infiltration. GO/KEGG pathway analyses revealed that Golgi vesicle transport and ER to Golgi vesicle-mediated transport might be associated with the effect of *GOLT1B* on cancer pathogenesis. *STRAP*, *INTS13*, and *ETNK1* were identified as the most relevant altered genes of *GOLT1B*, and their interactions were primarily linked to the regulation of the TGF-β receptor signaling pathway and EMT. Our study provides insights into the application of *GOLT1B* as a potential prognostic biomarker in several cancers in the context of immuno-oncology and contributes to the development of *GOLT1B*-targeting therapeutic strategies.

*GOLT1B* is a member of the GOT1 family. Limited studies have reported the oncogenic role of *GOLT1B* in human cancers.^[15,16]^ sCNA is a major source of genomic variations driving tumor evolution, and sCNA screening may identify prognostic biomarkers. The amplification of *GOLT1B* has been reported to be correlated with a worse prognosis in patients with resected LUAD.^[15]^ Consistent with previous studies, our study further confirmed that high expression levels of *GOLT1B* are linked to poor prognosis in many types of cancer. Copy number amplification was identified as the primary type of *GOLT1B* genetic alteration related to the prognosis of pan-cancer cases. Moreover, it has been reported that overexpression of genes from 12p11.2 to 12p12.1 is a feature of all TGCTs and the overexpression of genes from this region, particularly in non-seminomas and seminomas with amplification, might play a key role in driving TGCT progression.^[30]^ In our study, *STRAP* (chr12:15), *INTS13* (chr12:26), and *ETNK1* (chr12:22) were the most genetic alteration-related genes for *GOLT1B* (chr12:21), and were all located at 12p15-26, with amplification as the primary genetic alteration type. Furthermore, the highest amplification frequency of *GOLT1B* was identified in tumors of the reproductive system, particularly in nonseminomatous germ cell tumors (> 8%). Genetic alteration by amplification supports the prognostic potential of *GOLT1B*, and large cohort validations with complete clinical data across different cancers are warranted.

DNA methylation is a major form of epigenetic modification of DNA that regulates gene expression without altering the DNA sequence. DNA methylation generally suppresses gene expression by changing chromatin structure, DNA stability, and DNA conformation.^[2,31,32]^ Hyper-methylation within the promoter regions often leads to the silencing or inactivation of tumor suppressor genes in cancerous cells. In our study, the differential status of *GOLT1B* promoter methylation across diverse cancers was determined. The level of promoter methylation was significantly lower in BRCA, HNSC, LUAD, and PRAD primary tissues but higher in ESCA and PAAD primary tissues, compared to corresponding normal tissues. Meanwhile, the significant association of differential methylation status of probe cg07371838 and cg25816357 with prognosis in diverse cancer cases was observed. However, our pan-cancer analysis also showed that *GOLT1B* overexpression contributed negatively to prognosis in patients with ESCA and PAAD. Hyper-methylation of the *GOLT1B* promoter region failed to result in gene inactivation in ESCA. There are several potential mechanisms for this phenomenon. On one hand, the heterogeneity of different tumor pathological characteristics and the methylation status of different genetic locations may affect the level of gene expression and the direction of changes in different cancers. On the other hand, the cross-talk between epigenetic mechanisms and alternative RNA processing regulation plays critical roles in cell differentiation, organ development, and disease responses.^[33]^ *STRAP* was previously identified as a putative spliceosome-associated factor that regulates alternative splicing (AS) through preferred binding positions.^[34]^ In this study, *STRAP* was shown to have specific binding positions for *GOLT1B*, *INTS13*, and *ETNK 1* transcripts, respectively, suggesting the potential of *STRAP*-related AS regulatory mechanisms with these relevant genes. Considering the general as well as cancer type-specific and subtype-specific alterations in the AS of cancer, differentiated methylation status and levels of transcript and protein expression of *GOLT1B* across cancer types might be elucidated.^[35]^ The physiological relevance of the cross-talk between *GOLT1B* methylation and *STRAP*-regulated AS processing needs to be further investigated.

Despite some breakthroughs in cancer treatment, immunotherapy faces challenges in a successful application, while new targets and biomarkers are required to improve its efficacy.^[36,37]^ Dysregulations of diverse immune cells and components of the tumor microenvironment (TME) facilitate the immune escape of tumors, ultimately resulting in tumor proliferation, recurrence, and metastasis.^[38]^ GA plays an essential role in the trafficking, sorting, and modification of proteins and lipids in immune responses.^[7,39]^ The abnormal expression of GA-related proteins contributes to immunomodulator dysfunction, which significantly promotes cancer progression.^[40,41]^ Immunotherapy targeting Golgi resident proteins has entered the clinical trial stage.^[7]^ The *GOLT1B* expression was found to be closely associated with the tumor microenvironment score and infiltration of immune cells in BRCA.^[42]^ In our study, there was a positive correlation between *GOLT1B* expression and the infiltration level of CD4+ T lymphocytes, especially the T helper type 2 (Th2) subset, which is associated with poor prognosis in multiple cancers. As two predominant subsets of CD4+ T lymphocytes, Th1 and Th2 are characterized by the production of IFN-γ and interleukin (IL)-4 cytokines, respectively.^[43,44]^ It has been reported that the Th1 to Th2 immune polarity shift serves as an important feature for tumor progression.^[45]^ The cytokines secreted from Th2 cells, including IL-4, IL-5, and IL-13, have been confirmed to promote cancer development.^[44]^ T cells in the peripheral blood collected from patients with metastatic melanoma showed shifting from cytotoxic Th1 responses toward chronic inflammatory Th2 responses, indicated by elaboration of increased levels of IL-4 and decreased levels of IFN-γ.^[46]^ It is demonstrated that *GOLT1B* promotes the expression and facilitates the membrane localization of programmed death-ligand 2 (PD-L2), which plays an important role in Th2 immunity.^[16,47]^ Besides, the high expression of *GOLT1B* might inhibit GSK3β phosphorylation and obstruct the secretion of IFN-γ, further impeding the positive feedback regulation of IFN-γ on Th1 differentiation. Consistent with previous findings, our study showed the expression of *GOLT1B* positively correlated with the infiltration of Th2 cells. *GOLT1B* might induce immunological suppression by promoting Th1 to Th2 immune polarity shift, providing a new direction for immunotherapy. Additionally, the high expression of *GOLT1B* was also found to be positively associated with the infiltration of cancer-associated fibroblasts (CAFs), which were key components of the TME with diverse functions. Taken together, our results further confirmed the involvement of *GOLT1B* with the infiltration of different immune cells in cancers.

In addition, functional analysis revealed that the regulation of the TGF-β receptor signaling pathway and EMT were two critical pathways in the interaction between *GOLT1B* and related genes including *STRAP*, *INTS13*, and *ETNK1*. TGF-β has been reported to regulate immune responses and maintain immune homeostasis by regulating the proliferation, differentiation, and survival of multiple immune cell lineages.^[48]^ Moreover, TGF-β supports cancer growth and progression by activating tumor angiogenesis and CAFs while enabling the tumor to evade inhibitory immune responses.^[49]^ Our exploration of the signaling pathways mediated by *GOLT1B* genetic alterations revealed its involvement in EMT and TGF-β receptor signaling pathways, providing new evidence for the exploration of the regulatory mechanism of *GOLT1B* on immune cell infiltration.

## Conclusion

In summary, the present pan-cancer analysis, for the first time, offered a comprehensive understanding of the oncogenic role of *GOLT1B* across different cancers, demonstrating the potential of *GOLT1B* as a diagnostic biomarker and a target for cancer immunotherapy. Further studies are warranted to elucidate the specific regulatory mechanisms of *GOLT1B* in carcinogenesis to improve the efficacy of cancer diagnosis and the diverse application of immunotherapy.

## Supplementary Material

Supplementary material
