# Supplementary material for: A pan-cancer analysis of the oncogenic role of Golgi transport 1B in human tumors
Source: J Transl Int Med. 2023 Dec 20;11(4):433–48. doi: 10.2478/jtim-2023-0002 (PMC10732491; doi:10.2478/jtim-2023-0002)
Supplement: Supplementary file 1 — Supplementary material [file jtim-2023-0002_sm.pdf]

Supplementary Material

Table S1: Function list of associated molecules

Function	FDR	Genes in network	Genes in genome
regulation of transforming growth factor beta receptor signaling pathway	0.00036828	5	86
regulation of epithelial to mesenchymal transition	0.00036828	5	76
cellular response to transforming growth factor beta stimulus	0.00036828	6	169
transforming growth factor beta receptor signaling pathway	0.000410778	5	103
negative regulation of transmembrane receptor protein serine/threonine kinase signaling pathway	0.000410778	5	102
response to transforming growth factor beta	0.000410778	6	195
regulation of cellular response to transforming growth factor beta stimulus	0.000410778	5	104
epithelial to mesenchymal transition	0.000434269	5	108
regulation of cellular response to growth factor stimulus	0.000475618	6	222
transforming growth factor beta receptor binding	0.00112887	3	14
regulation of pathway-restricted SMAD protein phosphorylation	0.001231135	4	58
pathway-restricted SMAD protein phosphorylation	0.001294982	4	60
transmembrane receptor protein serine/threonine kinase signaling pathway	0.001338792	6	282
mesenchymal cell differentiation	0.003577738	5	185
regulation of transmembrane receptor protein serine/threonine kinase signaling pathway	0.005012526	5	201
ventricular septum development	0.005610916	3	27
phosphatidylethanolamine metabolic process	0.005909839	3	28
mesenchyme development	0.00593508	5	216
cardiac septum morphogenesis	0.019682002	3	43
cardiac ventricle morphogenesis	0.033243782	3	52
cardiac septum development	0.048711549	3	60
regulation of cardiac epithelial to mesenchymal transition	0.056968083	2	10
cardiac ventricle development	0.073634251	3	71
cardiac chamber morphogenesis	0.097012396	3	79

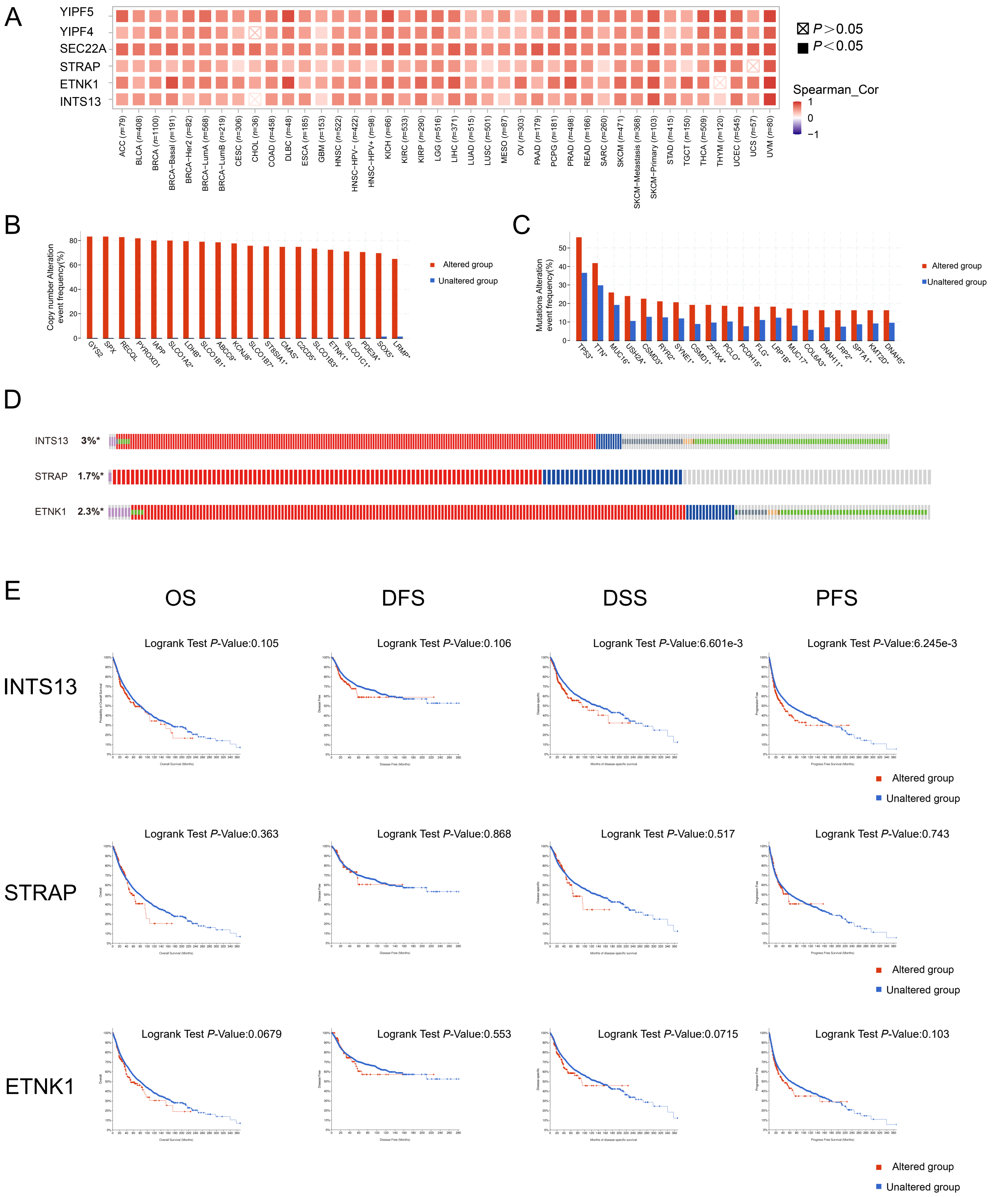

Figure S1: (A) The corresponding heatmap data of GOLT1B and intersection genes in the detailed cancer types. (B-C) Top20 genes with highest frequency in GOLT1B copy number alteration tumor groups and mutations alteration tumor groups of TCGA tumor. (D) Types of the genetic alteration of STRAP, INTS13 and ETNK1. (E) The potential correlation between the clinical survival prognosis of pan-cancer and genetic alteration of STRAP, INTS13 and ETNK1.
